# Ilizarov technique in an adolescent patient with progressive pseudorheumatoid dysplasia: A case report

**DOI:** 10.1097/MD.0000000000011375

**Published:** 2018-08-03

**Authors:** Ke Xiao, Tao Li, Yaping Jiang, Zheng Li, Qiankun Zhu, Zhihong Wu, Xisheng Weng

**Affiliations:** a Department of Orthopaedic Surgery, Peking Union Medical College Hospital, Peking Union Medical College, Beijing; b Department of Orthopaedic Surgery; c Department of Stomatology, The Affiliated Hospital of Qingdao University, Qingdao; d Department of Central Laboratory, Peking Union Medical College Hospital, Peking Union Medical College and Chinese Academy of Medical Sciences, Beijing, China.

**Keywords:** Ilizarov technique, joint deformity, progressive pseudorheumatoid dysplasia

## Abstract

**Rationale:**

Progressive pseudorheumatoid dysplasia (PPD) is a rare autosomal recessive inherited disease that causes severe systemic joint deformity and articular dysfunction in young patients.

**Patient concerns:**

Ilizarov technique treatment in PPD patients has never been reported before.

**Diagnoses:**

A 17-year-old male patient presented with a 10-year history of polyarthritis and 4-year history of progressive hip and knee pain and stiffness. Genetic testing for the WISP3 gene was done and showed compound heterozygous mutations: NM_198239.1 (WISP3):c.1064_1065dupGT (p.Gln356ValfsTer33) and NM_198239.1 (WISP3):c.643+2T > C.

**Interventions:**

Taking his young age into consideration, the Ilizarov external fixation technique was adopted for the treatment of the deformity in knees.

**Outcomes:**

One year after the operation, the improvement of joint deformity was satisfactory.

**Lessons:**

The Ilizarov technique is economical and less invasive, and most importantly, it can delay the possible arthroplasty. It gives young PPD patients with arthropathy an alternative treatment.

## 1 Introduction

Progressive pseudorheumatoid dysplasia (PPD) (MIM 208230), initially described by Wynne-Davies^[1]^ in 1982, is a rare genetic disease triggered by recessive gene mutations in *WISP3* (Wnt1-inducible signaling pathway protein 3; MIM 603400). PPD is characterized by progressive noninflammatory multiple arthropathy impairing mainly the articular cartilage. The symptoms commonly manifest between the age of 3 and 10 years with progressive joint pain and stiffness, and it is often misdiagnosed with juvenile rheumatoid arthritis by the onset, thus leading to prescription of useless anti-inflammatory and immunosuppressive medications. Despite the first symptoms appearing in early childhood, the diagnosis of PPD is often not made until late puberty when the patients already suffer from obvious joint and spine deformities.

Previous reports on PPD treatments have been rarely seen. A case of total-hip arthroplasty (THA) was reported in a 17-year-old female patient with PPD,^[2]^ and the 1-year follow-up was satisfactory. Just as the 17-year-old patient, most PPD sufferers begin to have severe hip and knee deformity and dysfunction in their adolescence. However, THA for patients at such young ages is quite controversial. Although the short-term feedback of PPD treated by THA is good, the long-term prognosis is in doubt because revision surgeries are highly unavoidable. In addition, most patients with PPD have osteoporosis because of *WIPS3* mutation, so the revision surgeries for them are risky and probably are needed for more than once in their lifetime. Taking this into account, the external fixation such as the Ilizarov technique is possibly an alternative for arthroplasty and can postpone joint replacement.

## 2 Case report

**Figure 1:**
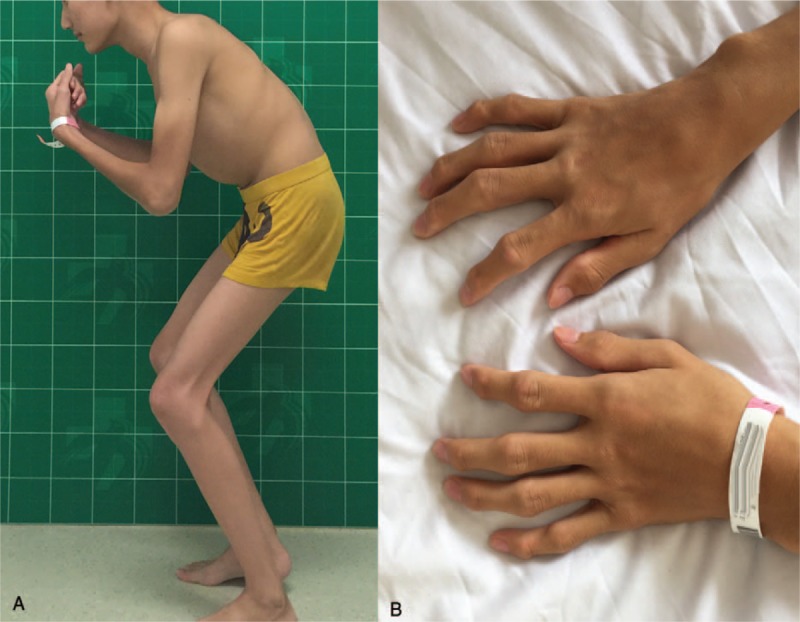
(A) Fixed flexion deformity of hips and knees. (B) Multiple interphalangeal joints were enlarged.

A 17-year-old male patient presented with a 10-year history of polyarthritis and 4-year history of progressive hip and knee pain and stiffness. His interphalangeal joints were first involved, and then the elbows, knees, and hips. He was misdiagnosed with developmental dysplasia of hips at the age of 9 in another hospital and received no treatment. Physical examination showed swaying gait with fixed flexion deformity of hips and knees (Fig. 1A). Multiple interphalangeal joints were enlarged (Fig. 1B). Impaired range of motion of his interphalangeal joints, elbows, and wrists was observed. Hip flexion-extension was 70° to −45° in the left and 80° to −40° in the right. Knee flexion-extension was 120° to −40° in the left and 120° to −45° in the right. No obvious scoliosis or thoracic kyphosis was noticed. Babinski sign was negative and the knee-jerk reflex was normal. Laboratory tests including blood cell count, erythrocyte sedimentation rate, C-reactive protein, rheumatoid factor, antistreptolysin O, and anticyclic cirullinated peptide antibodies were normal. Preoperative visual analog score (VAS) was 7 and the Harris hip score was 32.

**Figure 2:**
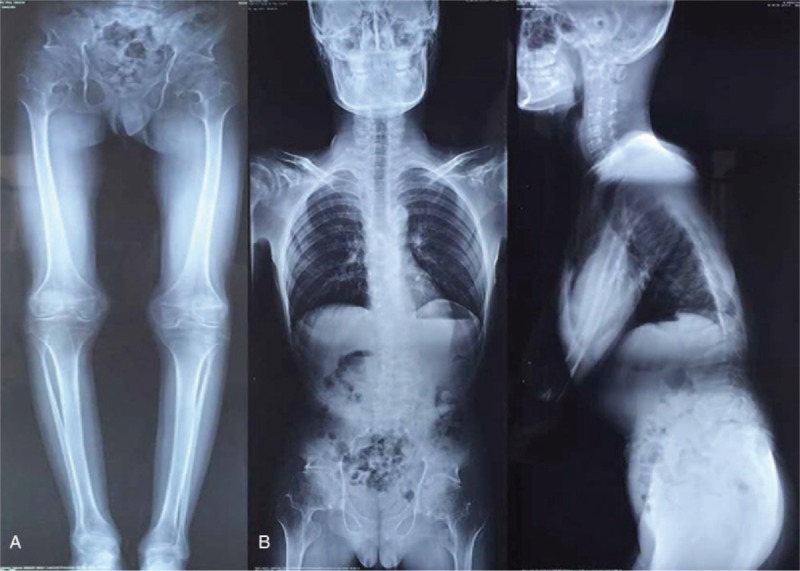
(A) Radiograph showed broadening width of the pubic symphysis, enlargement of femoral heads, narrowing hip joint space, enlargement of femoral condylar and narrowed joint space of knees. (B) Spinal X-rays showed platyspondyly, irregular end plates, and mild scoliosis.

Radiograph of pelvis showed broadening width of the pubic symphysis, enlargement of femoral heads, narrowed hip joint space, and osteoarthritis of hips. Radiograph of knees demonstrated enlargement of femoral condylar and narrowed joint space (Fig. 2A). Spinal X-rays revealed platyspondyly, irregular end plates, and mild scoliosis (Fig. 2B).

Written informed consent was obtained from the patient and the guardians. Genetic testing for the *WISP3* gene was done and showed compound heterozygous mutations: NM_198239.1 (WISP3): c.1064_1065dupGT (p.Gln356ValfsTer33) and NM_198239.1 (WISP3): c.643 + 2T > C. Combined with the patient's history, symptoms, physical signs, and genetic testing, the final definitive diagnosis is PPD.

Deduced from radiographs, the hip and knee joint space was decreased but still had room for remodeling by Ilizarov fixator, since this technique provides with secure fixation against persistent stress and tension, and allows for mild-to-moderate daily activities during treatment. Soft tissue around the contractural knee was stretched gradually is the rational for the Ilizarov techniques. Thus, we carefully worked out preoperative plans according to the condition of the patient and the characteristics of Ilizarov fixator. To deal with severe joint contractures, tenolysis, and soft tissue release needed to be performed, before imposing the fixator, by a 3-cm subinguinal longitudinal incision and another one at gastrocnemius tendon. After that, 4 steel rings should be placed sequentially at the mid-thigh, 5 cm above the knee cap, 5 cm below the knee cap, and above the ankle, and each ring required a piece of K-wire applied by percutaneous corticotomy cuts. Instead of being placed at a parallel pattern, the K-wires should be fixed at different angles, by a rotation of 45° at each plane, to form a stable 3-dimensional structure. We planned to extend the fixator 0.5 to 0.1 cm each day, adjusted by the tolerance of the patient, till a full extension of the lower extremity. Then, the same procedure should be applied to the opposite side, and a cast be used to consolidate the right limb for the same amount of time as dealing with the left limb.

**Figure 3:**
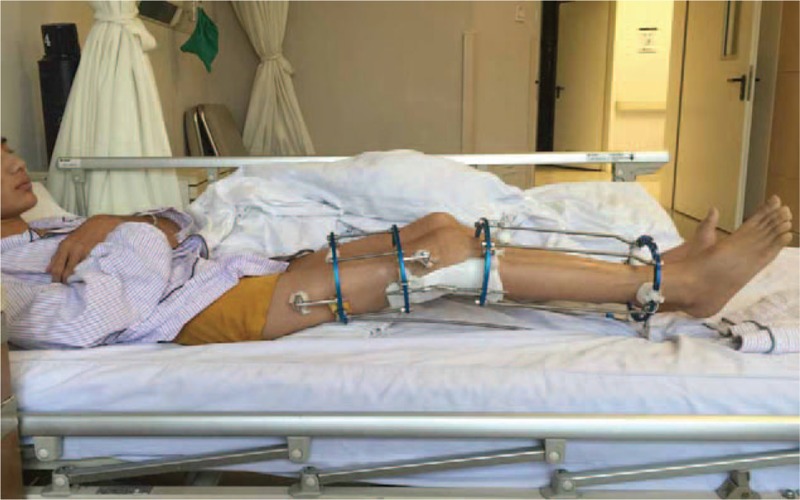
The Ilizarov external fixation apparatus was lengthened 5 to 10 mm/d.

**Figure 4:**
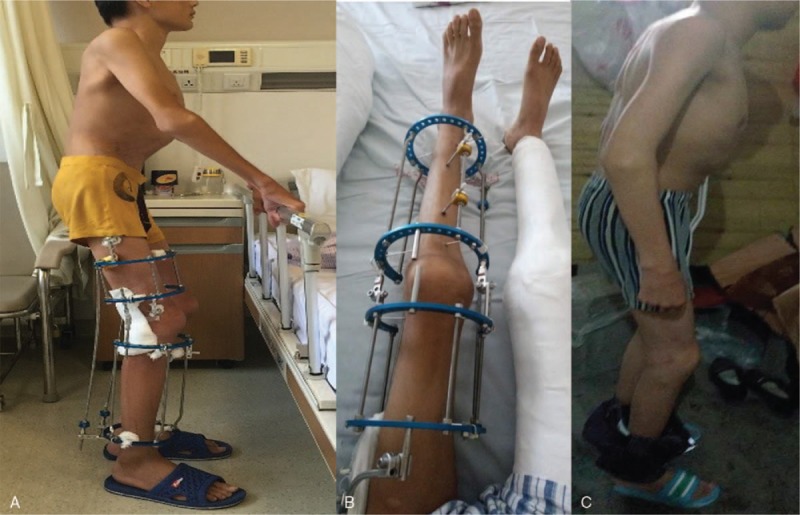
(A) Thirteen days after operation, flexion deformity of right hip and knee was much improved. (B) Thirty-six days after the operation, the Ilizarov technique was performed to his left hip and knee, and plaster cast for his right lower extremity was placed. (C) One year after operation, the improvement of hip and knee deformity was satisfactory.

At the first stage, we performed the surgery in his right hip and knee. The patient underwent the surgery under general anesthesia in a supine position. Firstly, the proximal end of rectus femoris muscle was released, and then the hamstring muscle and semitendinosus were released. After that, common peroneal nerve lysis was done. After checking the tension of common peroneal nerve, the Ilizarov external fixation apparatus was placed. After the operation, the Ilizarov external fixation apparatus was lengthened 5 to 10 mm/d (Fig. 3). Hip and knee passive movement was also performed every day. Thirteen days after the operation, the flexion deformity of his right hip and knee was greatly improved (Fig. 4A). There is no possibility of growth potential in this patient which can affect the result for closure of epiphysis.

Thirty-six days after the operation, the Ilizarov technique was performed to his left hip and knee by the same procedures and plaster cast for his right lower extremity was placed at the same time (Fig. 4B). One month after the stage 2 surgery, external fixation apparatus and plaster cast were removed. The follow-up time period is 1 year. One year after the operation, the improvement of joint deformity was satisfactory (Fig. 4C). The follow-up results are demonstrated in Table 1. There was no complication observed in this patient. The patient has provided informed consent for publication of the case.

**Table 1:**
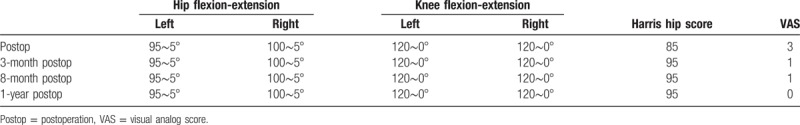
Follow-up results.

## 3 Discussion

The PPD is an autosomal recessive disorder that affects cartilage homeostasis and causes polyarthropathy.^[3]^ PPD is a rare disease that has been reported mostly in Western Asia and the Mediterranean, while the rest have been reported only sporadically.^[4,5]^ The patients with PPD can have no symptoms and signs during the infant period. Most patients with PPD begin to have manifestations between 3 and 10 years of age. Progressive symmetric polyarthralgia and stiffness affecting the interphalangeal joints, elbows, knees, hips, and ankles are the most common features, with joint range of motion impairment and deformities developing gradually. Patients with PPD may also have spinal involvement presenting as lumbar lordosis, thoracic kyphosis, or scoliosis.^[3,6,7]^ The first sign is interphalangeal joint pain and enlargement in 30% of PPD cases.^[8]^

At the beginning of the disease, the children were easily misdiagnosed as juvenile rheumatoid arthritis and were often treated in pediatrics, rheumatism immunology department, or orthopedic department. As a result, there are many unnecessary anti-inflammatory and immunotherapy. Because the disease is not caused by inflammation or immune disorders, anti-inflammatory and immunotherapy are ineffective. Therefore, PPD should be differentiated from juvenile rheumatoid arthritis to avoid unnecessary anti-inflammatory and immunotherapy.^[9]^

The PPD is caused by mutation of *WISP3* gene on chromosome 6q22.^[10]^*WISP3*, expressed in synoviocytes and chondrocytes, belongs to the CCN (cyr61, ctgf, nov) family that encodes proteins for cell growth and differentiation,^[11,12]^ playing a major part in bone growth and cartilage metabolism. Genetic analysis for *WISP3* is fundamental to the definite diagnosis of PPD. Because PPD pathogenesis is deficient of articular cartilage during growth and development, early diagnosis and treatment of supplemental cartilage components may slow down the progression of the disease and improve the prognosis of the disease.

At present, PPD can only be treated with symptomatic treatment. Theoretically, young patients can perform joint replacement surgery, which can effectively relieve pain and restore joint function. Total-knee arthroplasty (TKA) can be performed when the knee was involved in the disease, but in fact, TKA in PPD is rarely reported at present.^[2]^ Previous study reported THA treatment in a young patients with PPD and the 1-year follow-up was satisfactory. However, THA for teenage patients with arthropathy is quite controversial because revision surgery is highly unavoidable and it may be needed for several times. This is especially worth pondering for patients with PPD because many of them are teenagers with arthropathy and osteoporosis.

The Ilizarov method introduced by G.A. Ilizarov in the 1960s has developed immensely.^[13]^ The surgical interventions of Ilizarov techniques are not aggressive, spare tissues, and involve little blood loss. The Ilizarov method has been applied for the management of a great variety of bone diseases and conditions, such as severe open and closed fractures, congenital and acquired limb discrepancy, bone defects, and osteomyelitis.^[14]^

It was approved that the Ilizarov method is an efficient tool which can be successfully applied in the treatment of patients with the knee flexion contracture.^[15]^ For some patients with PPD with hip and knee deformity, the external fixation such as the Ilizarov technique is possibly an alternative for arthroplasty and can postpone joint replacement. The Ilizarov external fixation apparatus was lengthened 5 to 10 mm/d. Hip and knee passive movement was also performed every day. In this process, the soft tissue around the joint can be gradually loosened and opened, and the function of joint flexion and extension can be gradually restored. Thirteen days after the operation, the flexion deformity of his right hip and knee was greatly improved.

## 4 Conclusion

In summary, we reported a 17-year-old patient with PPD with the Ilizarov technique. He acquired satisfactory motion range improvement of hip and knee joints. Compared with THA, the Ilizarov technique is economical and less invasive, and most importantly, it can delay the possible arthroplasty. As far as we know, this is the first report of Ilizarov technique in patients with PPD. It gives young patients with PPD with arthropathy an alternative treatment. The shortcoming of our case is that the follow-up duration is not sufficient to observe the long-term prognosis.

## Author contributions

**Conceptualization:** Tao Li.

**Data curation:** Zheng Li.

**Methodology:** Xisheng Weng.

**Resources:** Qiankun Zhu.

**Visualization:** Zhihong Wu.

**Writing – original draft:** Ke Xiao.

**Writing – review & editing:** Yaping Jiang.
